# Nociceptive Sensitization by Activation of Protease-Activated Receptor 2 in a Rat Model of Incisional Pain

**DOI:** 10.3390/brainsci11020144

**Published:** 2021-01-22

**Authors:** Kanta Kido, Norika Katagiri, Hiromasa Kawana, Shigekazu Sugino, Masanori Yamauchi, Eiji Masaki

**Affiliations:** 1 Department of Anesthesiology, Kanagawa Dental University Hospital, Yokosuka, Kanagawa 2388570, Japan; katagiri@kdu.ac.jp; 2 Department of Oral and Maxillofacial Implantology, Kanagawa Dental University Hospital, Yokosuka, Kanagawa 2388570, Japan; kawana@kdu.ac.jp; 3 Department of Anesthesiology and Perioperative Medicine, Tohoku University School of Medicine, Sendai, Miyagi 9808575, Japan; sugino@ruby.ocn.ne.jp (S.S.); yamauchi@med.tohoku.ac.jp (M.Y.); 4 Department of Anesthesiology, International University of Health and Welfare Hospital, Nasushiobara, Tochigi 3292763, Japan; ejmasaki@gmail.com

**Keywords:** postoperative pain, protease-activated receptor 2, PAR-2, tryptase, mast cell

## Abstract

Postoperative pain and consequent inflammatory responses after tissue incision adversely affects many surgical patients due to complicated mechanisms. In this study, we examined whether activation of protease-activated receptor 2 (PAR-2), which is stimulated by tryptase from mast cells, elicits nociception and whether the PAR-2 antagonist could reduce incisional nociceptive responses in vivo and in vitro. The effects of a selective PAR-2 antagonist, N3-methylbutyryl-N-6-aminohexanoyl-piperazine (ENMD-1068), pretreatment on pain behaviors were assessed after plantar incision in rats. The effects of a PAR-2 agonist, SLIGRL-NH_2_, on nociception was assessed after the injection into the hind paw. Furthermore, the responses of C-mechanosensitive nociceptors to the PAR-2 agonist were observed using an in vitro skin–nerve preparation as well. Intraplantar injection of SLIGRL-NH_2_ elicited spontaneous nociceptive behavior and hyperalgesia. Local administration of ENMD-1068 suppressed guarding behaviors, mechanical and heat hyperalgesia only within the first few hours after incision. SLIGRL-NH_2_ caused ongoing activity in 47% of C-mechanonociceptors in vitro. This study suggests that PAR-2 may support early nociception after incision by direct or indirect sensitization of C-fibers in rats. Moreover, PAR-2 may play a regulatory role in the early period of postoperative pain together with other co-factors to that contribute to postoperative pain.

## 1. Introduction

Postoperative pain is caused by multiple processes including inflammatory, neuropathic and ischemic components [1,2,3,4]. Multiple analgesic methods and drugs have been applied to reduce postoperative pain [5,6]; nevertheless, these management strategies do not always improve the pain due to their undesirable adverse effects [6]. Various mechanisms of postoperative pain have been investigated, including excitation, sensitization and inhibition of the peripheral and central nervous system [1,7,8]. In peripheral tissue, many inflammatory mediators from immune cells or resident cells involving neutrophils, platelets, macrophages and mast cells, contribute to peripheral nociceptive sensitization after tissue injury [9,10].

Mast cells are one of the resident immune cells in connective tissue. They play crucial roles in the early phase of immune responses and they are called “gatekeepers” [11], because they mainly reside near the nerve fibers in the subcutaneous tissue [12]. Mast cells release a variety of mediators which produce nociception on primary afferent neurons by binding to receptors which are expressed on the afferents [11,13,14,15]. A few recent studies have investigated the association between mast cells and postoperative pain. Oliveira et al. described that preventing degranulation of mast cells reduced spontaneous pain and mechanical allodynia after plantar incision in mice [16,17], and we also previously reported that mast cell stabilization by cromoglycate promoted antinociceptive effects in mice [18]. However, the antagonism of typical mediators like serotonin and histamine has shown weak efficacy in a surgery-induced pain in rodents [16,19,20], suggesting that other mediators could participate in the pain process.

When mast cells degranulate and release tryptase, this produces nociception by binding to the protease activated receptor-2 (PAR-2) on primary afferents [11,13]. Recent studies have shown that the activation of PAR-2 is related to some painful diseases including arthritis and inflammatory bowel disease [21,22,23]. PAR-2 is one of the G protein-coupled receptors which is expressed on primary sensory neurons, and the agonist triggers release of substance P and calcitonin gene-related peptide (CGRP) from peripheral nerve ending [24]. However, the part of PAR-2 activation in painful processes is still not understood. Only one study examined the effects of PAR-2 antagonist pretreatment on postoperative pain in mice [17]. In the study, antagonism of PAR-2 produced a short-lasting effect in preventing postoperative pain in vivo, and surgery increased tryptase activity in tissue perfusates, suggesting that tryptase released from mast cell was a candidate to activate PAR-2 on nociceptive terminals and contributed to nociception caused by surgery. However, little is known about the effects of PAR-2 activation on nociceptors. In this study, we examined rats and we tested whether the selective PAR-2 antagonist N3-methylbutyryl-N-6-aminohexanoyl-piperazine (ENMD-1068) could reduce incisional nociception, and whether the injection of a PAR-2 agonist SLIGRL-NH_2_ elicited pain in vivo. We also examined whether the agonist activates mechanosensitive primary afferents in an in vitro skin–nerve preparation.

## 2. Materials and Methods

### 2.1. General

All experimental procedures were reviewed and approved by the Committee of Ethics on Animal Experiments of Kanagawa Dental University (protocol No. 18-035) and Institutional Laboratory Animal Care and Use Committee of Tohoku University (protocol No. 2015-009). Adult male Sprague–Dawley rats (250–300 g; SLC, Hamamatsu, Japan) were used in this study. Rats were housed under a 12-h light–dark schedule. Food and water were available ad libitum.

### 2.2. Drug Preparation

Selective PAR-2 antagonist, N3-methylbutyryl-N-6-aminohexanoyl-piperazine (ENMD-1068) was purchased from Enzo Life Sciences (Farmingdale, NY, USA) and solved in phosphate buffered saline (PBS). PAR-2-activating peptide (PAR-2-AP, SLIGRL-NH_2_), corresponding to the tethered ligand of rat PAR-2, was obtained from Sigma-Aldrich Japan (Tokyo, Japan).

### 2.3. Intraplantar Drug Administration

To investigate the effects of local subcutaneous injection of PAR-2 antagonist on the incisional model, injection (ENMD-1068; 10 mM, 100 µL) was performed 30 min before paw incision using a microsyringe with a 30-gauge needle and under light sevoflurane anesthesia. The control group received 100 µL PBS in the same manner. Guarding behavior and responses to thermal and mechanical stimulation were measured after plantar incision. The PBS solution was used as the control.

In a separate series of experiments, PAR-2-activating peptide (SLIGRL-NH_2_; 1 mM, 100 µL) was injected into the plantar surface of the hind paw subcutaneously using a 30-gauge needle and a microsyringe in a total volume of 100 μL. For the control group, PBS was injected in the same manner. To assess spontaneous pain behaviors, drugs were injected into the middle of the tori of the paw using gentle restraint without anesthesia. Spontaneous pain and responses to heat and mechanical stimuli were measured after injection. The investigator performing the behavioral data was blinded to drug.

### 2.4. Plantar Hind Paw Incision

The surgery was performed as described previously [25]. Briefly, rats were anesthetized with 3 to 4% sevoflurane in air via a nose cone. A 10-mm longitudinal incision was made with a #11 surgical blade through the skin, fascia, and the plantar flexor digitorum brevis muscle. Blunt-curved forceps were then inserted through the incision into the muscle to further divide and retract the muscle. The wound was then closed with two subcutaneous mattress sutures with 5-0 nylon on a P-1 needle (Ethicon, USA) and covered with 3% tetracycline ointment from Sun pharma Japan (Tokyo, Japan). The control sham-operated rats underwent anesthesia and sterile preparation and no incision.

### 2.5. Behavioral Studies

#### 2.5.1. Spontaneous pain behavior

After injection of PAR-2-AP or other solutions, rats were immediately returned into the box on the mesh floor and the ipsilateral hind paw was observed for 30 min. Data were collected in 5-min bins after PAR-2-AP injection. Time spent shaking, licking, lifting and biting the hind paw was recorded.

#### 2.5.2. Guarding Behavior

A pain score was determined to evaluate guarding pain behaviors as described previously [26]. In brief, rats were acclimated to the testing environment for two or three hours per day three days before testing. A baseline test was then performed one day before surgery. To perform guarding behavior testing, rats were individually placed into a clear plastic cage top (21 × 26 × 16 cm) on a small plastic mesh floor (grid 8 × 8 mm). Both ipsilateral and contralateral hind paws of each animal were closely observed during a one-minute period repeated for every five minutes for one hour. A guarding pain score of 0, 1 or 2 was given, depending on the position in which each paw was found during one minute. A score of 0 was recorded when full weight bearing with the incised region was blanched or distorted by the mesh, 1 was recorded when the wound area just touched the mesh without blanching or distortion and 2 for the wound area completely off of the mesh surface. The sum of the 12 scores (0–24) recorded during one hour for each paw was obtained.

#### 2.5.3. Heat Sensitivity

Each rat was placed on a glass floor covered with a clear plastic cage top (21 × 26 × 16 cm) and acclimated. Heat withdrawal latencies were assessed by applying a focused radiant heat source (IITC-390 Plantar Test Analgesia Meter, IITCR Life Science Instruments, Woodland Hills, CA, USA) underneath a glass floor on the middle of the incised or injected area. The intensity of the heat was set to produce withdrawal latency in control rats of 10–15 s. Each rat was tested three times, at an interval of 10 min. The latency to evoke a withdrawal was determined with a cut-off value of 20 s. The average of three trials was used to obtain paw withdrawal latency.

#### 2.5.4. Mechanosensitivity

Each rat was individually placed on a plastic mesh floor covered with a clear plastic cage top (21 × 26 × 16 cm) and allowed to acclimate. Withdrawal response to punctate mechanical stimulation was determined using calibrated mono filaments (Touch Test Sensory Evaluator, Stoelting, IL, USA) applied underneath the cage through to an area adjacent to the wound or the middle of tori of the paw of the injected drug. Each filament was applied once starting with 10 mN and continuing until a withdrawal response occurred or 250 mN was reached. When no response to the 250 mN filaments was observed, the next filament 522 mN was recorded. Each rat was tested three times with at least a 5-min interval between withdrawal responses. The lowest force from the three tests producing a response was determined as the withdrawal threshold [27].

### 2.6. In Vitro Single Fiber Recordings

#### 2.6.1. Preparation

The rat in vitro skin–nerve preparation has been previously described [28]. Briefly, the rats were euthanized with carbon dioxide, and medial and lateral plantar nerves and their innervated glabrous hind paw skin were dissected and removed. The isolated preparation was immediately placed in an organ bath, which was continuously superfused with synthetic interstitial fluid (SIF; 107 mM of NaCl, 26.2 mM of NaHCO_3_, 9.64 mM of sodium gluconate, 5.5 mM of glucose, 7.6 mM of sucrose, 3.48 mM of KCl, 1.67 mM of NaH_2_PO_4_, 1.53 mM of CaCl_2_, and 0.69 mM of MgSO_4_), which had been oxygenated with a mixture of 95% O_2_ and 5% CO_2_. The temperature of the solution was maintained at 32 °C ± 0.5 °C. The skin was placed epidermal side down and the plantar nerve was drawn through a small hole to the recording chamber, which was filled with paraffin oil. The nerve was placed on a mirror, desheathed, and filaments were repeatedly teased using sharpened forceps and placed on a platinum electrode under microscopic view, until single-unit activity could be recorded. Neural activity was amplified (DAM50, Harvard Apparatus, Holliston, MA), filtered, and displayed using standard techniques. Amplified signals were led to an oscilloscope and an audiomonitor and fed into a PC computer via a data acquisition system (spike2/CED1401 program, Cambridge Electronic Design, Cambridge, UK).

#### 2.6.2. Identification of Afferents

We focused on the mechanosensitive C-fiber nociceptors in this study. Receptive fields were found by probing with a fire polished glass rod in the subdermal side of skin. Only units that were clearly distinguished (signal-to-noise ratio greater than 2:1) were further studied.

#### 2.6.3. Ongoing Activity

The receptive field was identified using a hollow metal cylinder with silicone grease to prevent leakage from the bath into the receptive field. After a 3-min baseline recording, the SIF solution inside the ring was removed with a syringe. Then, either SLIGRL-NH_2_ 100 µM or PBS-vehicle in-200 µL volume was applied to the receptive field and the activity was recorded for 3 min. The afferent was considered activated by SLIGRL-NH_2_ if activity of at least 0.1 impulse/s was produced if ongoing background activity was absent during baseline recording, or if ongoing activity was present an increase of at least two standard deviations greater than the ongoing background activity.

#### 2.6.4. Heat Stimulation

The effect of SLIGRL-NH_2_ on heat response was then tested using a ramp-shaped thermal stimulus to the receptive field using a feedback-controlled Peltier thermal stimulator with a circular probe (diameter: 1 mm, intercross-2000 N, Intercross, Tokyo, Japan). After the effect of SLIGRL-NH_2_ or PBS-vehicle on ongoing activity was complete, a computer-controlled heat ramp was delivered starting from 32 to 47 °C over 30 s. The peak temperature, 47 °C, was determined to avoid potential tissue damage of higher temperatures. Each fiber was examined only once with either SLIGRL-NH_2_ or PBS-vehicle for heat responses and tested in separate experiments for mechanical stimuli preventing heat stimuli from sensitizing the afferent responses. Action potentials were counted during the heat stimulus (for 30 s) and for 20 s after the peak temperature. The temperature that induced the first action potential was considered as heat threshold under no background ongoing activity. If background activity was present, the threshold was defined by the temperature during heating that increased background activity at least two standard deviations greater than the baseline (10 s, 1 s bin). Ongoing activity of background was subtracted from total action potential numbers during the recording period, postulating that background activity continued during the stimulation.

#### 2.6.5. Mechanical Stimulation

Mechanosensitivity was assessed using a servo force-controlled mechanical stimulator (Series 300C Dual Mode Servo System; Aurora Scientific, Aurora, Ontario, Canada). A flat-ended cylindrical metal probe (tip diameter, 0.7 mm) attached to the tip of the stimulator arm was applied to the most sensitive point of the receptive field with no force generated.

For mechanical testing, each fiber was examined twice, once each with PBS-vehicle and subsequently with SLIGRL-NH_2_. First, the SIF solution inside the ring was removed and PBS-vehicle solution with 200-µL volume was applied. After 2 min of PBS application, a computer-controlled ascending series of square force stimuli was applied to the receptive field (5, 10, 20, 40, 80 and 120 mN; 2 s duration; 60 s intervals). After testing PBS with application, the solution inside the ring was gently replaced with SIF solution a few times for washing out. Then, the SIF solution inside the ring was replaced with 100 µM SLIGRL-NH_2_ with 200-µL volume. After 2 min of drug application, the same series of mechanical stimulations was applied to the receptive field. The mechanical force which evoked the first action potential was determined as the mechanical threshold under no background activity. If background activity was present, mechanical threshold was determined by the force that increased background activity at least two standard deviations greater than the average background for baseline recording. Ongoing activity of background was subtracted from the total number of action potentials during mechanical stimuli, postulating that background activity continued during the stimulation.

#### 2.6.6. Conduction Velocity

The conduction velocity was evaluated at the end of the experiment. The conduction velocity of each fiber was determined by a bipolar electrode to induce action potentials in the afferent in the most mechanosensitive spot in the receptive filed (0.2–1.0 Hz, 5–20 V, 0.5–2.0 ms duration). Then the distance between the recording electrode (conduction distance) and the receptive field was divided by the latency of the action potential. Afferent fibers conducting slower than 2.5 m/s were classified as C-fibers, those conducting between 2.5 and 24 m/s as Aδ-fibers, and those conducting faster than 24 m/s as Aβ-fibers. Units were classified as mechanosensitive nociceptors on the basis of their graded response throughout the innocuous and noxious range of mechanical force stimuli. Rapidly adapting fibers were excluded. A-delta fibers were also recorded. However, the number was too few to analyze (5 fibers for ongoing activities, 4 for mechanical responses and 2 for heat responses).

### 2.7. Statistical Analysis

The Kolmogorov–Smirnov test was used for testing of normality. Guarding pain score and withdrawal latency to heat data were analyzed using 2-way analysis of variance (ANOVA), followed by 1-way ANOVA and the post hoc t-test with Bonferroni correction. Spontaneous pain behaviors were analyzed using 2-way ANOVA. The differences between groups were evaluated using two-way repeated measures ANOVA followed by the post hoc Sidak’s multiple comparisons test. Mechanical behavioral data were analyzed the using Friedman test. Between group differences were subsequently analyzed by the Mann–Whitney rank sum test (two groups) to compare drug at each time point vs. PBS-vehicle. All behavioral data are presented as means ± standard error of the mean (SEM). The chi-square test was performed to analyze prevalence of PAR-2-AP responsive and heat responsive units between groups for electrophysiological data. Heat response threshold and total action potentials during heat stimulation were analyzed using the Mann–Whitney rank sum test. The mechanical stimulus–response functions between groups were analyzed using two-way ANOVA with repeated measures. Data are presented as means ± SEM. A *p*-value less than 0.05 was considered statistically significant. All tests were performed with GraphPad Prism software (GraphPad, San Diego, CA, USA).

## 3. Results

### 3.1. Behavioral Studies

#### 3.1.1. Effect of PAR-2 Agonist, SLIGRL-NH_2_ on Pain Behaviors

**Figure 1:**
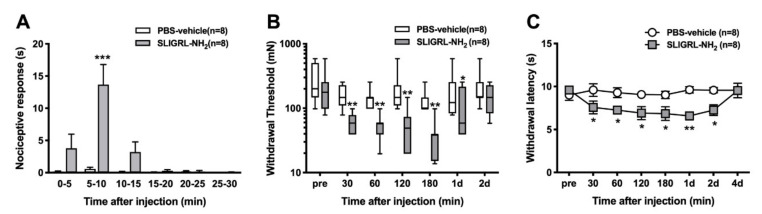
Effects of protease-activated receptor 2 (PAR-2) agonist SLIGRL-NH_2_ on nociceptive behavior, withdrawal threshold to mechanical stimuli, and withdrawal latency to heat after injection. (**A**) Time course of the nociceptive behavior including shaking, licking, lifting and biting of the hind leg after intraplantar injection of SLIGRL-NH_2_ or phosphate buffered saline (PBS) (*n* = 8 to both groups). Data were collected in 5-min bins from eight animals in each group. Data show the mean and standard error of the mean (SEM) of nociceptive time in 5-min bins. Statistical analysis used 2-way ANOVA with repeated measures on one factor followed by Bonferroni’s post hoc test for comparing the mean of the nociceptive time at each 5-min point. (**B**) Withdrawal threshold to mechanical stimuli applied to the hind paw. The results are presented as the median with range, interquartile range (box) and 10th and 90th percentile for eight rats in each group (*n* = 8 to both groups). A non-parametric Friedman’s test followed by Dunn’s post hoc test for between group comparisons at each time point were used. (**C**) Withdrawal latency to heat stimuli. The results are presented as the mean and SEM. Two-way ANOVA with repeated measures on one factor followed by Bonferroni’s post hoc test for comparing the mean withdrawal latency at each time point between groups (*n* = 8 to both groups) was performed. * *p* < 0.05, ** *p* < 0.01, *** *p* < 0.001 compared with the PBS-vehicle group at each time point.

PBS-vehicle produced very little spontaneous pain behavior (Figure 1A). There was a greater effect after injection of 1 mM 100 µL of SLIGRL-NH_2_, which produced nociceptive behavior from 5 min to 10 min compared with PBS-vehicle (0.6 ± 0.2 vs. 13.7 ± 3.1, *p* < 0.001; *n* = 8 per group). PBS-vehicle did not increase the mechanical responses compared with pre injection (Figure 1B). However, intraplantar injection of PAR-2-AP significantly induced mechanical hyperalgesia for one day after injection. Treatment of PBS did not decrease the withdrawal latency to radiant heat compared with pre injection (Figure 1C). Intraplantar injection of PAR-2-AP significantly induced the heat hypersensitivity compared with PBS-vehicle from 30 min to 2 days after injection.

#### 3.1.2. Effect of PAR-2 Antagonist, ENMD-1068 on Pain Behaviors after Plantar Incision

**Figure 2:**
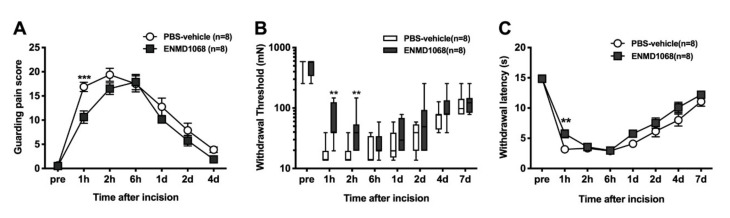
Effects of PAR-2 antagonist N3-methylbutyryl-N-6-aminohexanoyl-piperazine (ENMD 1068) on pain behaviors of rats after incision. (**A**) Guarding pain behavior. The results are presented as the mean and SEM for eight rats in each group (*n* = 8 to both groups). Statistical analysis used 2-way ANOVA with repeated measures on one factor followed by Bonferroni’s post hoc t-test for comparing the mean cumulative pain score at each time point between groups. (**B**) Withdrawal threshold to mechanical stimuli applied to the hind paw. The results are presented as the median with interquartile range (box) and 10th and 90th percentile for eight rats in each group (*n* = 8 to both groups). A non-parametric Friedman’s test followed by Dunn’s post hoc test for between- group comparisons at each time point were used. (**C**) Withdrawal latency to heat stimuli. The results are presented as the mean and SEM. Statistical analysis used 2-way ANOVA with repeated measures on one factor followed by Bonferroni’s post hoc test for comparing the mean withdrawal latency at each time point between groups (*n* = 8 to both groups). ** *p* < 0.01, *** *p* < 0.001 compared with the vehicle group at each time point. POD = postoperative day.

In the group of incised rats treated with PBS-vehicle, the guarding pain score increased from one hour to two days after incision compared with baseline (Figure 2A). Pretreatment with ENMD-1068 decreased the guarding behavior by paw incision at 1 h compared with PBS. This was the only significant decrease in cumulative pain score (10.6 ± 1.3 vs. 16.9 ± 1.0, *p* < 0.001, PBS; *n* = 8 per group). Paw incision induced a decrease in mechanical withdrawal threshold a during the 4 days following incision as displayed in Figure 2B. Intraplantar injection of ENMD-1068 increased the mechanical withdrawal threshold from 1 h to 2 h after incision. In the PBS-treated group, the mean withdrawal latency to radiant heat decreased from sec s 14.9 ± 0.6 to 3.2 ± 0.2 1 h after incision (Figure 2C). One hour after incision, ENMD-1068 increased the withdrawal latency compared to the PBS control (5.7 ± 0.7; *p* < 0.01). ENMD-1068 did not affect heat withdrawal latency thereafter.

### 3.2. Single Fiber Recording

**Figure 3:**
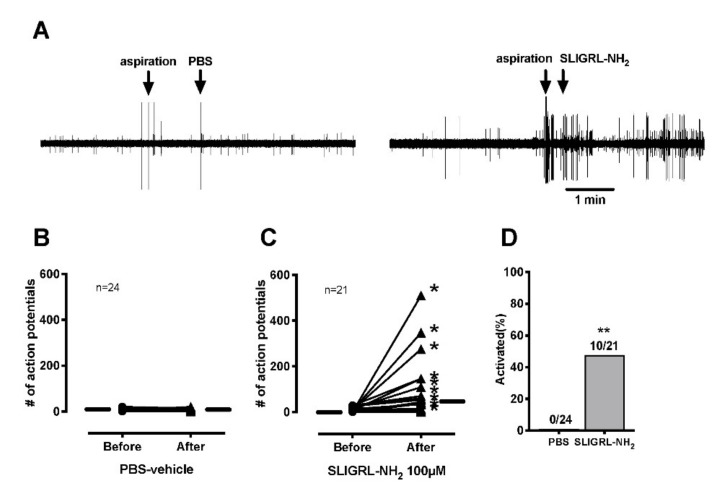
Effects of PAR-2 agonist SLIGRL-NH_2_ or vehicle on ongoing activity of mechanonociceptive C-fibers. Each fiber was tested with one application, either PBS or SLIGRL-NH_2_. (**A**) Example recordings of from 2 different C-fibers before and after application of PBS-vehicle or 100 µM SLIGRL-NH_2_. (**B**,**C**) Ongoing activities of C-fibers before and after exposure to 100 µM SLIGRL-NH_2_ or PBS-vehicle. Each line on the graphs represents a single unit. Asterisks indicate activation of nociceptors by drug application. Small horizontal lines in each graph indicate median values. (**D**) The proportion of C-fibers responsive to application of SLIGRL-NH_2_ or PBS-vehicle. A total of 24 units for PBS and 21 units for SLIGRL-NH_2_ were used.

**Figure 4:**
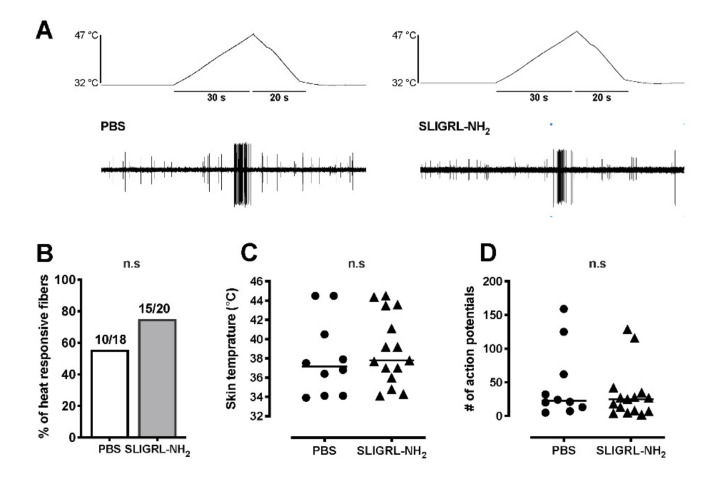
Effects of PAR-2 agonist SLIGRL-NH_2_ on heat responses of mechanonociceptive C-fibers. (**A**) Example recordings of C-fibers after application of either PBS-vehicle or 100 µM SLIGRL-NH_2_. Each fiber was tested with one application, either PBS or SLIGRL-NH_2_. (**B**) Proportion of C-fibers responsive to heat after application of SLIGRL-NH_2_ or PBS-vehicle. (**C**,**D**) Heat responses of C-fibers after exposure to SLIGRL-NH_2_ or PBS-vehicle. Heat thresholds (**C**) and total action potentials for 50 s during heat application (**D**). Each symbol represents a single unit.

**Figure 5:**
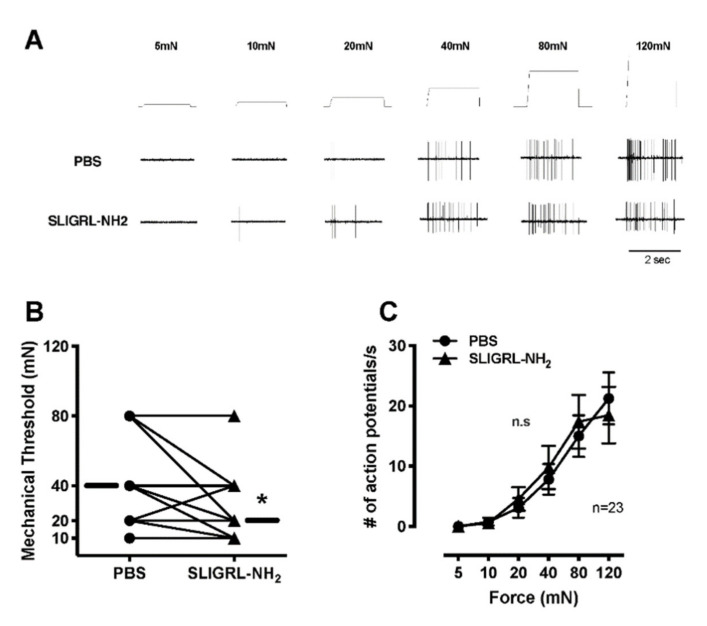
Effects of PAR-2 agonist SLIGRL-NH_2_ on mechanical responses of mechanonociceptive C- fibers. Each fiber was tested with application of PBS followed by SLIGRL-NH_2_. (**A**) Example recordings of C-fibers after application of PBS-vehicle and 100 µM SLIGRL-NH_2_. (**B**) Mechanical thresholds of C-fibers after application of PBS-vehicle and SLIGRL-NH_2_. Each line represents a single unit. The horizontal lines in each graph indicate median values. (**C**) Mechanical stimulus response function of C-fibers after application PBS-vehicle and SLIGRL-NH_2_. Data are presented as means ± SEM. * *p* < 0.05.

PBS-vehicle did not increase the ongoing activity of C-fibers (Figure 3A,B). SLIGRL-NH_2_ increased the ongoing activity of 10 of 21 C-mechanonociceptive fibers (47.6%) (Figure 3D). The median increase in activity after application of SLIGRL-NH_2_ was 36 imp/3 min (Figure 3A,C). For heat, SLIGRL-NH_2_ did not increase the proportion of heat responsive C-fibers (15/20, 75.0%; *p* = 0.31) compared to PBS-vehicle (10/18, 55.6%) (Figure 4A,B). SLIGRL-NH_2_ did not affect the heat threshold or the magnitude of the heat response (Figure 4C,D). The median mechanical thresholds of C-fibers after exposure to SLIGRL-NH_2_ was lower than PBS-vehicle (20 vs. 40 mN; *p* < 0.05) (Figure 5A,B). The number of action potentials at each force was not different between SLIGRL-NH_2_ and PBS-vehicle (Figure 5C). SLIGRL-NH_2_ had also no effect on the mechanical responses of C-fibers (Figure 5A).

## 4. Discussion

We tested whether protease-activated receptor 2 (PAR-2), influenced incisional nociception in vivo in rats. In addition, we examined the ability of the PAR-2 agonist SLIGRL-NH_2_ to activate or sensitize primary afferent in the in vitro skin–nerve preparation. The main findings of this study are that (i) activation of PAR-2 using an agonist, SLIGRL-NH_2_ induced nociceptive pain behavior and increased heat and mechanical responsiveness, (ii) blockade of PAR-2 using a selective antagonist, ENMD-1068, reduced incision-induced guarding pain behavior, mechanical and heat hypersensitivity, (iii) SLIGRL-NH_2_ caused C-nociceptor activation but did not affect the heat and mechanical responsiveness in the in vitro skin–nerve preparation. These results indicate that the activation of PAR-2 by incision induced spontaneous nociceptive effects, alongside mechanical and heat hypersensitivity; however, the effects of PAR-2 activation alone were limited in vitro. Taken together, other co-factors could be needed to contribute to sustained activation and sensitization of nociceptors.

Previously, it was demonstrated that intraplantar administration of cromoglycate, a mast cell stabilizer, reduced the pain responses expressed as guarding pain and mechanical hyperalgesia in a mouse postoperative pain model [18]. Thus, stabilization of mast cells at an operation site might reduce postoperative pain caused by various mediators from the mast cell degranulation.

Moreover, in work by others, the PAR-2 agonist, SLIGRL-NH_2_, induced nociceptive pain behaviors immediately after the injection and significantly increased mechanical and heat hyperalgesia within a few days after administration in mice. To our knowledge, only one study examined a role for PAR-2 in non-evoked pain [29]. Tillu et al. described that intraplantar injection of PAR-2 agonist in mice caused an increase in spontaneous pain score, and the duration of the pain state was less compared with mechanical hypersensitivity. They concluded that PAR-2 agonist directly evoked spontaneous pain behavior [29].

Studies by others support a role for PAR-2 in nociception. Vergnolle et al. described that proteinases and selective PAR-2 agonists stimulated nociceptive neurons and caused hyperalgesia to both heat and mechanical stimuli in mice [30]. They described that PAR-2 was expressed on dorsal root ganglion (DRG) neurons which also expressed CGRP and Substance P, and activation of PAR-2 directly signaled to the neurons and stimulated the release of both CGRP and Substance P from C-fibers in peripheral tissues and in the spinal cord. In our in vitro study, a PAR-2 agonist directly induced ongoing activity of C-fiber but had little effect on the heat and mechanical responses of the primary afferents. These results, taken together, indicate that PAR-2 activation might be one component of early postoperative spontaneous ongoing pain and promotes release of the peptides including Substance P or CGRP from DRG and spinal cord, which lead to heat and mechanical hyperalgesia. Furthermore, other pronociceptive mediators or other receptors’ activation, including TRPV1, [31] affected by PAR-2 activation, could play crucial roles in sustaining or potentiating pain after surgery. In fact, protons, prostaglandins, interleukins and nerve growth factor (NGF) have been demonstrated to be released later after the incision [32,33,34,35,36]. PAR-2 activated by mast cell tryptase could be activated early in postoperative pain.

In this study, blockade of PAR-2 using a selective antagonist, ENMD-1068 briefly decreased incision-induced guarding pain, mechanical and heat hypersensitivity, highlighting the contribution of peripheral PAR-2 to early incisional pain (Figure 2). Preventing mast cells from degranulating (with Compound 48/80 prior to incision or pretreatment of mast cell stabilizer) markedly reduced postoperative hyperalgesia in mice [16,18]. Meanwhile, antagonism of histamine or serotonin receptors only partially reduced the postoperative mechanical hyperalgesia and non-evoked pain in mice [16]. Therefore, it was considered that other mediators from mast cells must be involved. Only one study focused on the effects of PAR-2 antagonist on postoperative pain in a preclinical study [17]. Oliveira et al. described that pretreatment with the selective PAR-2 antagonist ENMD-1068, and the selective tryptase inhibitor gabexate, reduced postoperative mechanical hyperalgesia and spontaneous nociception in a mouse model of postoperative pain.

Although pretreatment with ENMD-1068 reduced guarding pain score, mechanical and heat hyperalgesia after plantar incision in our study, the efficacy might be limited to the early period after surgery. One of the reasons may be that the activity of tryptase (or other mediators from mast cells as well) was detected within 10 min but not 30 or 60 min after plantar surgery [17]. In fact, Oliveira et al. examined the effect of posttreatment with ENMD-1068, PAR-2 antagonist, on mechanical hyperalgesia after plantar incision. They showed that the antagonist did not alter the intensity of postoperative nociception [17]. Furthermore, it was considered that the PAR-2-induced pain pathway after incision is not sufficient to block pain related behaviors, and that other mediators contribute [1,37].

In our study, SLIGRL-NH_2_, a PAR-2 agonist caused ongoing activity which was increased in 47% of C-mechanonociceptors in vitro skin–nerve preparation but had little effect on the heat and mechanical responsiveness of the afferents. Previous studies have shown that PAR-2 was expressed by approximately 60% of neurons in the dorsal root ganglia, where it was found in small-diameter, nociceptive neurons containing Substance P and CGRP [24,38]. Supporting the behavioral tests in this study, PAR-2 activation directly excited the C-mechanosensitive nociceptors, however, did not sensitize the primary nociceptors to heat and mechanical stimuli. These results suggested that (i) the nociception of the primary afferents by PAR-2 activation resulting in spontaneous activity might contribute to heat and mechanical responses via central sensitization or (ii) the nociception by PAR-2 activation might require other circulating molecules that affected sensory neuron excitability, because these might have been “washed out” in our in vitro recording conditions.

Recently, the cooperative interaction between PAR-2 activation and transient receptor potential (TRP) ion channels including TRPV1, TRPV4 and TRPA1 has been clarified in nociceptive signaling and pain [39,40,41]. PAR-2 is highly co-expressed with TRPV1 receptors in the DRG neurons [31], and PAR-2 activation leads to TRPV1 sensitization to endogenous agonist [42,43]. PAR-2 activation might be a trigger which enhanced TRP channels or other receptors leading to the nociception of the primary afferents [29].

There were several limitations to this study. First, we did not examine the dose dependency of the effect of PAR-2 antagonist, ENMD 1068, and agonist, SLIGRL-NH_2_. Both optimal doses were based on previous studies [17,30]. In the study by Oliveira et al. [17], intraplantar administration of 100 nmol/20 µL ENMD-1068 (33 µg/paw ≒ 1 µg/g body weight) was effective for blockade of PAR-2 in mice and hence 10 mM/100 µL ENMD-1068 (330 µg/paw ≒ 1 µg/g body weight) was injected intraplantarly in rats in our study. Similarly, in the study by Vergnolle et al. [30], intraplantar administration of 1~10 µg/paw SLIGRL-NH_2_ was effective to induce hyperalgesia in mice, and hence 1 mM/100 µL (77.1 µg/paw) was injected in rats. Second, mechanosensitive-Aδ fibers were not assessed, because insufficient numbers of Aδ were collected for analysis. However, PAR-2 was expressed by approximately 60% of neurons in the dorsal root ganglia, where it was found in small-diameter, mostly nociceptive C-fibers containing Substance P and CGRP [30]. Therefore, a focus on C-fibers is warranted.

## 5. Conclusions

Our results suggest that PAR-2 may support early nociception after incision by sensitization of C-nociceptors in rats. Moreover, PAR-2 may contribute to the development of postoperative pain together with other inflammatory mediators or channel activation. Further studies are needed to determine other co-factors which enhance and sustain postoperative pain.

## Data Availability

The data presented in this study are available on request from the corresponding author.
